# Characterization of two SGNH family cell death-inducing proteins from the horticulturally important fungal pathogen *Botrytis cinerea* based on the optimized prokaryotic expression system

**DOI:** 10.1186/s43897-024-00086-3

**Published:** 2024-03-07

**Authors:** Xiaokang Zhang, Zhanquan Zhang, Tong Chen, Yong Chen, Boqiang Li, Shiping Tian

**Affiliations:** 1 Key Laboratory of Plant Resources, Institute of Botany, Chinese Academy of Sciences, Beijing, 100093 China; 2 University of Chinese Academy of Sciences, Beijing, 100049 China

**Keywords:** *Botrytis cinerea*, Cell death-inducing protein, SGNH family hydrolases, Plant-fungus interaction

## Abstract

*Botrytis cinerea* is one of the most destructive phytopathogenic fungi, causing significant losses to horticultural crops. As a necrotrophic fungus, *B. cinerea* obtains nutrients by killing host cells. Secreted cell death-inducing proteins (CDIPs) play a crucial role in necrotrophic infection; however, only a limited number have been reported. For high-throughput CDIP screening, we optimized the prokaryotic expression system and compared its efficiency with other commonly used protein expression systems. The optimized prokaryotic expression system showed superior effectiveness and efficiency and was selected for subsequent CDIP screening. The screening system verified fifty-five candidate proteins and identified two novel SGNH family CDIPs: BcRAE and BcFAT. *BcRAE and BcFAT* exhibited high expression levels throughout the infection process. Site-directed mutagenesis targeting conserved Ser residues abolished the cell death-inducing activity of both BcRAE and BcFAT. Moreover, the transient expression of BcRAE and BcFAT in plants enhanced plant resistance against *B. cinerea* without inducing cell death, independent of their enzymatic activities. Our results suggest a high-efficiency screening system for high-throughput CDIP screening and provide new targets for further study of *B. cinerea*-plant interactions.

**Supplementary Information:**

The online version contains supplementary material available at 10.1186/s43897-024-00086-3.

## Core

In this study, we optimized the prokaryotic expression system for screening the cell death-inducing protein (CDIP). The optimized screening system identified two novel CDIPs in the SGNH family of B. cinerea, namely BcFAT and BcRAE. Sufficient BcFAT and BcRAE can trigger plant cell death, which depends on their enzymatic activities. The transient expression of BcFAT and BcRAE in plants was found to induce plant resistance against B. cinerea without inducing cell death, which is independent of enzyme activity.

## Gene and accession numbers

Information for the genes in this article can be found in the National Center for Biotechnology Information (NCBI) database, accession numbers:BcFAT (BCIN_07g03280), BcRAE (BCIN_02g07100).

## Introduction

*Botrytis cinerea*, the causal agent of the notorious plant disease gray mold, is one of the most devastating plant pathogens worldwide. *B. cinerea* has a remarkably wide host range and infects more than 1000 plant species, including almost all fruit and vegetable crops (Yigal et al. 2016). Moreover, *B. cinerea* attacks horticultural crops not only in the field but also during postharvest storage, causing an annual economic loss of over $10 billion (Chen et al. 2023b). Due to its substantial economic impact and scientific importance, *B. cinerea* ranks second among the top ten plant fungal pathogens worldwide (Dean et al. 2012). Currently, the control of *B.cinerea* is still mainly dependent on chemical fungicides in agricultural production. However, fungicide-resistant strains have emerged in the field, and the overuse of fungicides has led to increasing concerns about environmental pollution and chemical residues (Poveda et al. 2020). Thus, alternative strategies are urgently needed to control *B. cinerea*.

Bio-sourced antifungal substances have attracted broad attention recently for their prominent properties, such as being non-toxic, readily available, and environmentally friendly (Chen et al. 2023a). To date, a series of bio-sourced substances have been reported with remarkable antifungal activities and promising potential for controlling *B. cinerea* (Wang et al. 2020; Cui et al. 2021; Sun et al. 2021; Li et al. 2022a; Zhang et al. 2023). An in-depth understanding of the pathogenic mechanism of *B. cinerea* is conducive to discovering novel antifungal substances and promoting the practical application of biogenic substances. In addition, controlling essential pathogenic factors or inducing disease resistance in postharvest fruits or vegetables are alternative strategies to reduce the dependence on chemical fungicides, which also requires an in-depth understanding of the pathogenic mechanisms of *B. cinerea* and the *B. cinerea*-plant interaction mechanisms (An et al. 2015; Li et al. 2020; Gu et al. 2021; Li et al. 2022b; Lu et al. 2023; Yang et al. 2023). Therefore, a deep understanding of the pathogenic mechanism of *B. cinerea* is of great significance for developing effective control strategies.

*B. cinerea* has been reported to employ multiple mechanisms to promote necrotrophic infection, such as the release of phytotoxic metabolites as well as the secretion of cell wall-degrading enzymes and cell death-inducing proteins (CDIPs) (Veloso and van Kan 2018; Zhang et al. 2021a). Among them, CDIPs trigger hypersensitive response-like cell death in plants, and the modes of action of several CDIPs have been elucidated. For example, SPL1, an abundantly secreted cerato-platanin family protein, interacts with plant PR1 and induces cell death in plants (Zhang et al. 2017; Yang et al. 2018a). The abundantly secreted glycoprotein BcIEB1 was reported to interact with PR5 and trigger plant cell death, possibly as a pathogen-associated mode (González et al. 2017). The xyglucanase BcXYG1 causes intense plant necrosis through a SOBIR1/BAK1-related pathway, and its cell death-inducing activity is independent of its xyglucanase activity (Zhu et al. 2017). The recently reported BcCrh1 is secreted into the plant apoplast and translocated into the plant cell, triggering cell death and the immune response (Bi et al. 2021). These results indicate that CDIPs activate the plant immune system and trigger cell death critical to infection. However, only a few CDIPs have been reported among more than 500 secreted proteins of *B. cinerea* (Zhu et al. 2017, 2022; Yang et al. 2018b; Bi et al. 2021; Jeblick et al. 2023). Recent research has shown that the secretome of multiple knockout mutants generated by CRISPR/Cas9 that lack 12 reported CDIPs retains substantial phytotoxic activity, suggesting that there are still unknown CDIPs contributing to necrosis and virulence (Leisen et al. 2022). To further identify novel CDIPs, an efficient high-throughputput characterization system is necessary. Prokaryotic, eukaryotic, and *Agrobacterium*-mediated transient expression systems are three commonly used heterologous protein expression systems. *Agrobacterium*-mediated transient expression system is a widely used CDIP screening and characterization system. However, unstable protein expression efficiency and unquantifiable protein expression levels may affect the screening and characterization of CDIPs. The prokaryotic expression system has a high protein expression level and efficiency. However, the frequent formation of insoluble inclusion bodies of prokaryotic expression limits its expression effect and application. The eukaryotic expression system has post-translational protein modifications with low interference from protein impurities. Nevertheless, which system is more suitable for screening and characterizing CDIPs has not yet been compared.

Here, we optimized the prokaryotic protein expression system, promoted the formation of soluble proteins, and established a CDIP screening system based on the optimized prokaryotic expression. We further compared the screening efficiency and effectiveness of three protein expression systems. The optimized prokaryotic expression system exhibited superior screening effects and was selected as the final screening system. With the help of the screening system, we screened CDIPs from 55 candidate proteins and identified two novel SGNH family CDIPs. The SGNH superfamily is a large hydrolase family named after four highly conserved residues (serine, glycine, asparagine, and histidine) and comprises a diverse range of carbohydrate-modifying enzymes (Anderson et al. 2022; Lescic Asler et al. 2017). SGNH family members have been reported to contribute to plant immunity (Oh et al. 2005; Kwon et al. 2009; Kim et al. 2013). However, there are few reports on the role of fungal SGNH proteins in plant-fungus interactions. Therefore, we also conducted preliminary functional studies on these two SGNH family CDIPs in this study.

## Results

### Optimization of the prokaryotic expression system

**Fig. 1:**
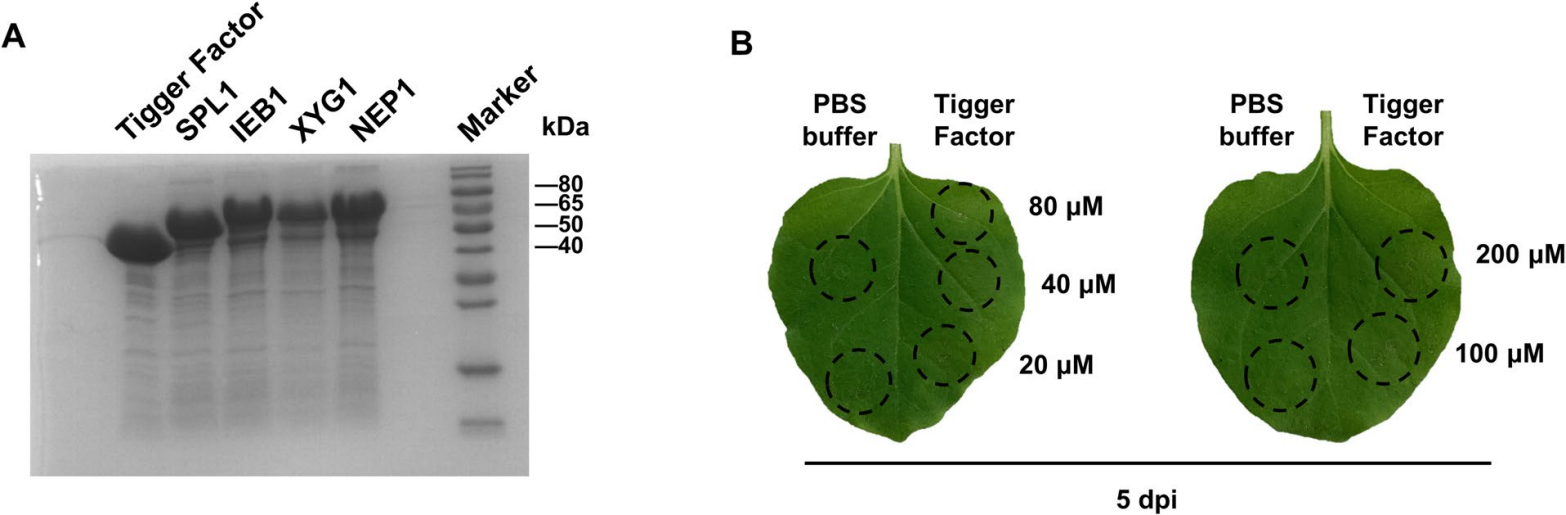
Optimization of the prokaryotic expression system. **A** Expression of soluble recombinant cell death-inducing proteins (CDIPs) utilizing the optimized prokaryotic expression system. The reported CDIPs BcIEB1 (BCIN_15g00100), BcNEP1 (BCIN_06g06720), BcSPL1 (BCIN_03g00500), and BcXYG1 (BCIN_03g03630) were recombinantly expressed with trigger factor (TF) using the prokaryotic expression system, and the TF was expressed as a control. The supernatant of disrupted cells was used for protein purification. **B** The impact of TF on plants. TF at different concentrations were infiltrated into tobacco leaves, and 10 mM phosphate-buffered saline (PBS) was used as the negative control. Photos were taken 5 d after infiltration. Black dashed circles indicate infiltrated sites

Most *B. cinerea*-secreted proteins have small molecular weights and are prone to form insoluble inclusion bodies during prokaryotic expression. To optimize the prokaryotic expression system for CDIP screening, we explored the expression effects of different expression vectors and expression conditions. The results showed that the CDIPs expressed by the pET30a vector at 37 °C and 15 °C were only present in the sediment (Fig. S1A and B), indicating that the protein formed insoluble inclusion bodies under these expression conditions. With the help of the pCold-TF vector harboring the molecular chaperone trigger factor (TF), we induced the expression of TF-CDIP recombinant proteins at 15 °C overnight. The TF-CDIPs mainly existed in the supernatant and existed in the precipitation (Fig. S1C). After further purification of the supernatant, we obtained TF-CDIP recombinant proteins with prominent bands (Fig. 1A). Moreover, in the subsequent protein expression, most proteins were present in the supernatant (Fig. S2). To further verify the effect of TF on plants, we infiltrated tobacco with different concentrations of TF. The results showed that 20–200 μM TF had no obvious impact on plants (Fig. 1B). These results indicate that overnight induction at 15 °C with the help of the molecular chaperone TF is a suitable condition for prokaryotic expression of CDIP.

### Comparison of protein-expressing systems for screening CDIPs

**Fig. 2:**
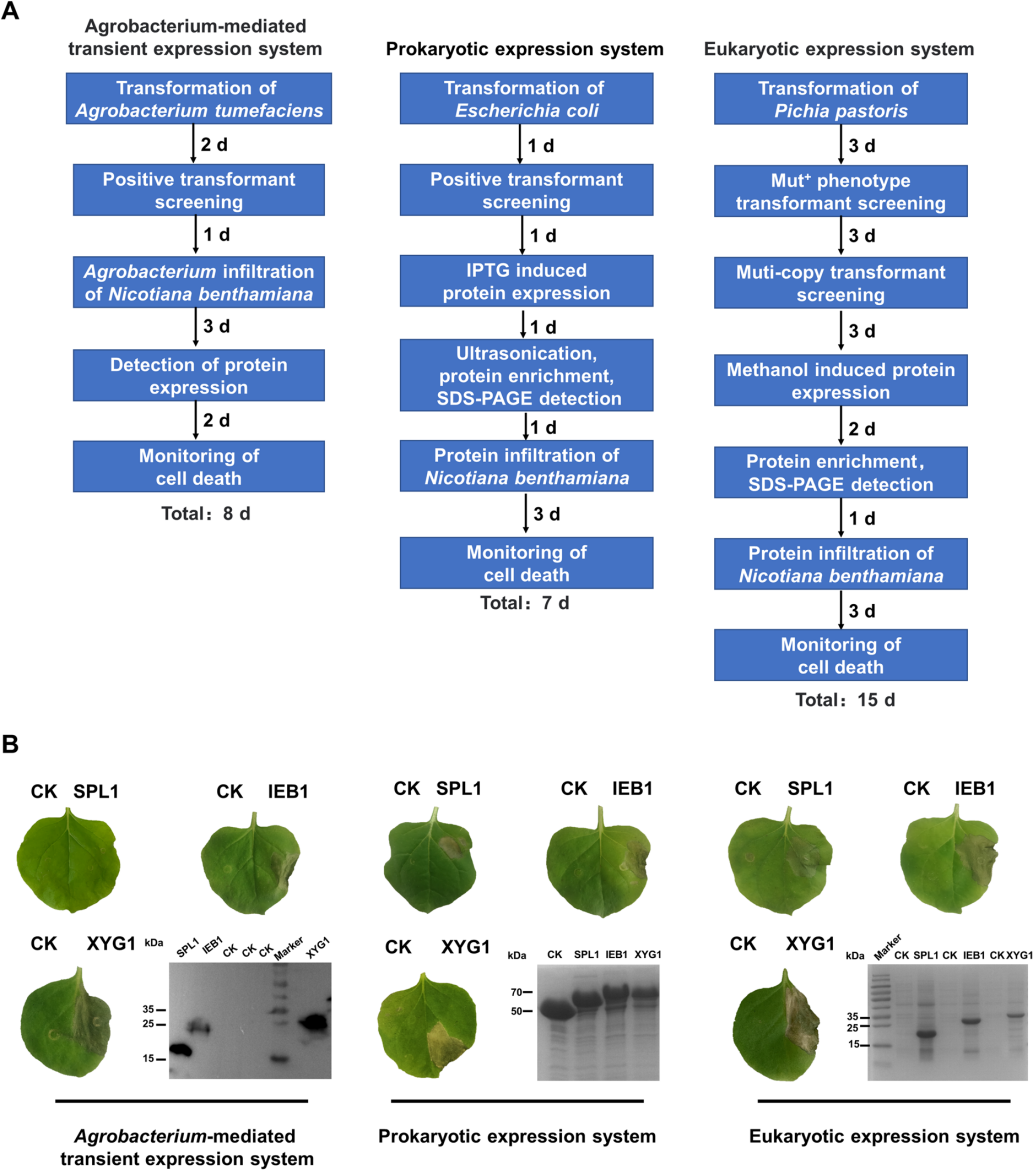
Comparison of different protein expression systems. **A** Flow chart of the protein expression processes of different systems. **B** The reported CDIPs BcIEB1, BcSPL1, and BcXYG1 were used to test the screening effects of different expression systems. *Agrobacterium tumefaciens* for transient expression was infiltrated into tobacco leaves at OD600 = 0.5. Photos were taken five days after infiltration. Western blotting (WB) was performed to verify the expression of proteins in plants. SDS-PAGE verified the proteins expressed by the eukaryotic and prokaryotic expression systems. Proteins expressed by the eukaryotic and prokaryotic expression systems were infiltrated into tobacco leaves at a concentration of 50 μM. Photos were taken 3 d after infiltration. Empty vectors were used to express control proteins (CK), respectively

High-throughput screening of CDIPs requires an effective and efficient screening system. To determine the appropriate screening system, we compared the efficiency and effectiveness of the eukaryotic, prokaryotic, and *Agrobacterium*-mediated transient expression systems on CDIP screening. Previously reported CDIPs, including BcSPL1 (Frías et al. 2011), BcXYG1 (Zhu et al. 2017), and BcIEB1 (Frías et al. 2016), were expressed by different protein expression systems. The necrosis-inducing activities were then verified in tobacco leaves. Figure 2A shows the protein expression processes of the three protein expression systems. Compared with prokaryotic and *Agrobacterium*-mediated transient expression systems, the eukaryotic system is time-consuming and labor-intensive. As shown in Fig. 2B, all proteins expressed by the eukaryotic and prokaryotic expression systems induced necrosis in plants. However, the transiently expressed BcSPL1 failed to cause necrosis, and the WB results showed that the protein was successfully expressed in plants. The above results suggest that eukaryotic and prokaryotic expression systems are more effective than the transient expression system for CDIP screening. The *Agrobacterium*-mediated transient expression system is a commonly used screening system for necrosis-inducing proteins. Our results indicate that the transient expression system probably misses important CDIPs during screening. Moreover, the eukaryotic expression system is time-consuming and labor-intensive, thus unsuitable for high-throughput screening. The prokaryotic expression system exhibited outstanding screening efficacy, high expression levels, and low time and labor costs. Therefore, the prokaryotic expression system was chosen for the subsequent screening of CDIPs.

### Screening of CDIPs by the optimized prokaryotic expression system

**Fig. 3:**
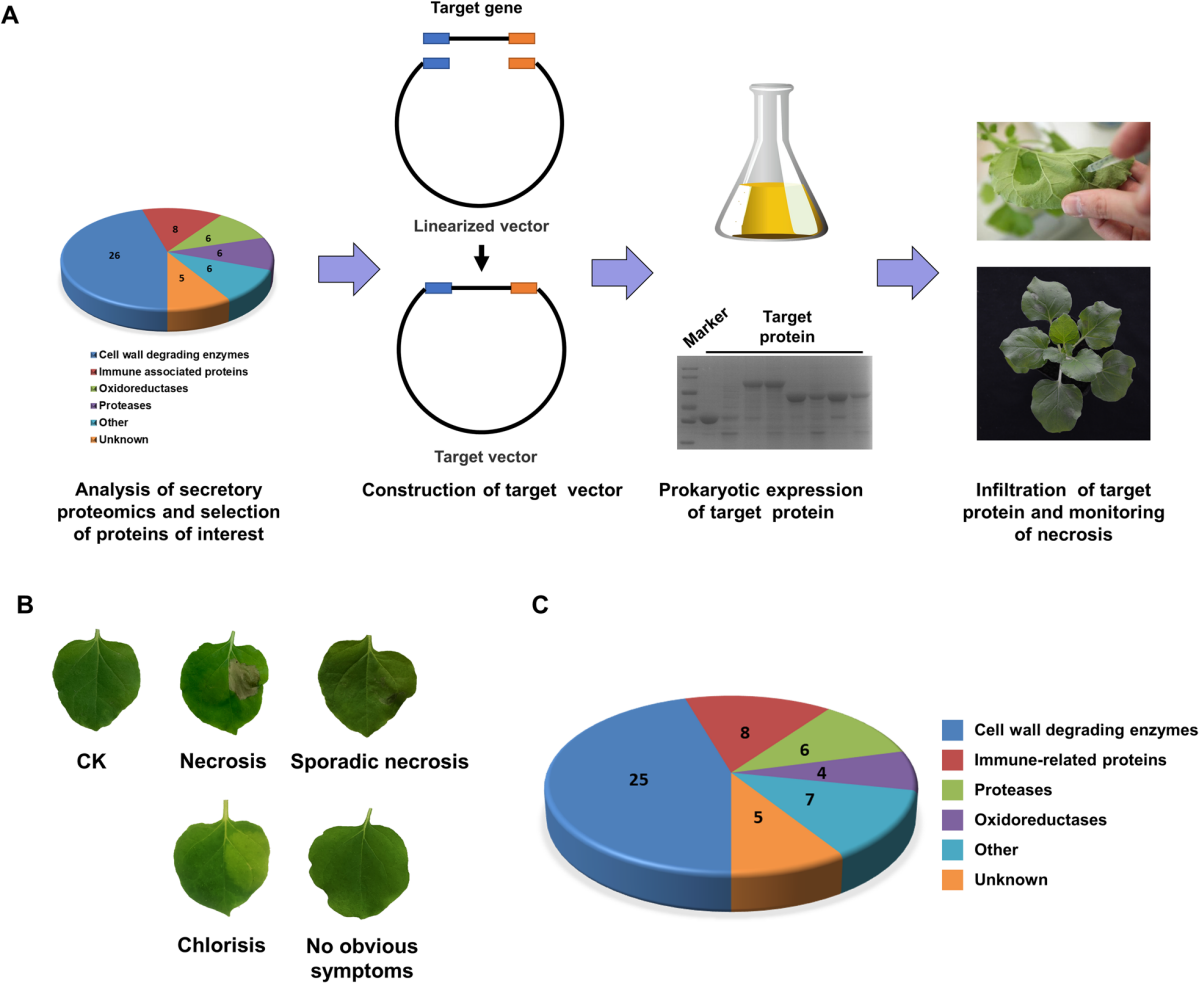
Screening of CDIPs by the optimized prokaryotic expression system. **A** Flowchart of screening CDIPs with the optimized prokaryotic expression system. Proteins of interest were preliminarily selected from secretomes. ORFs of the genes of interest without a signal peptide were homologously recombined into the pCold-TF vector. *Escherichia* coli harboring the recombined vectors were induced by 0.5 mM IPTG to express proteins overnight at 15 ℃, and SDS-PAGE was performed to examine the protein expression. The supernatant from disrupted cells was used for protein purification. Purified proteins were infiltrated into tobacco leaves at the same concentration, and the necrosis-inducing activities were monitored. **B** Representative *Nicotiana benthamiana* leaves with different cell death symptoms. Photos were taken 3 d after infiltration. **C** Functional classification of candidate proteins

The flowchart of screening CDIPs with the optimized prokaryotic expression system is shown in Fig. 3A. With the help of the optimized prokaryotic expression system, we further screened CDIPs of *B. cinerea*. We first selected 55 candidate proteins from the reported secretomes. Detailed information about the candidate proteins, including the gene ID number, amino acid number, function, and signal peptide, is shown in Table S1. The candidate proteins were expressed through the prokaryotic expression system, and proteins verified by SDS-PAGE (Fig. S2) were infiltrated into *Nicotiana benthamiana* leaves to detect cell death symptoms. The cell death symptoms were classified into necrosis, sporadic necrosis, chlorosis, and no obvious necrosis. Representative leaves are shown in Fig. 3B. The candidate proteins were classified by their functions, mainly into cell wall-degrading enzymes, immune-related proteins, oxidoreductases, proteases, and other proteins of unknown function (Fig. 3C).

### Two SGNH hydrolases, BcRAE and BcFAT, lead to cell death in plants

**Fig. 4:**
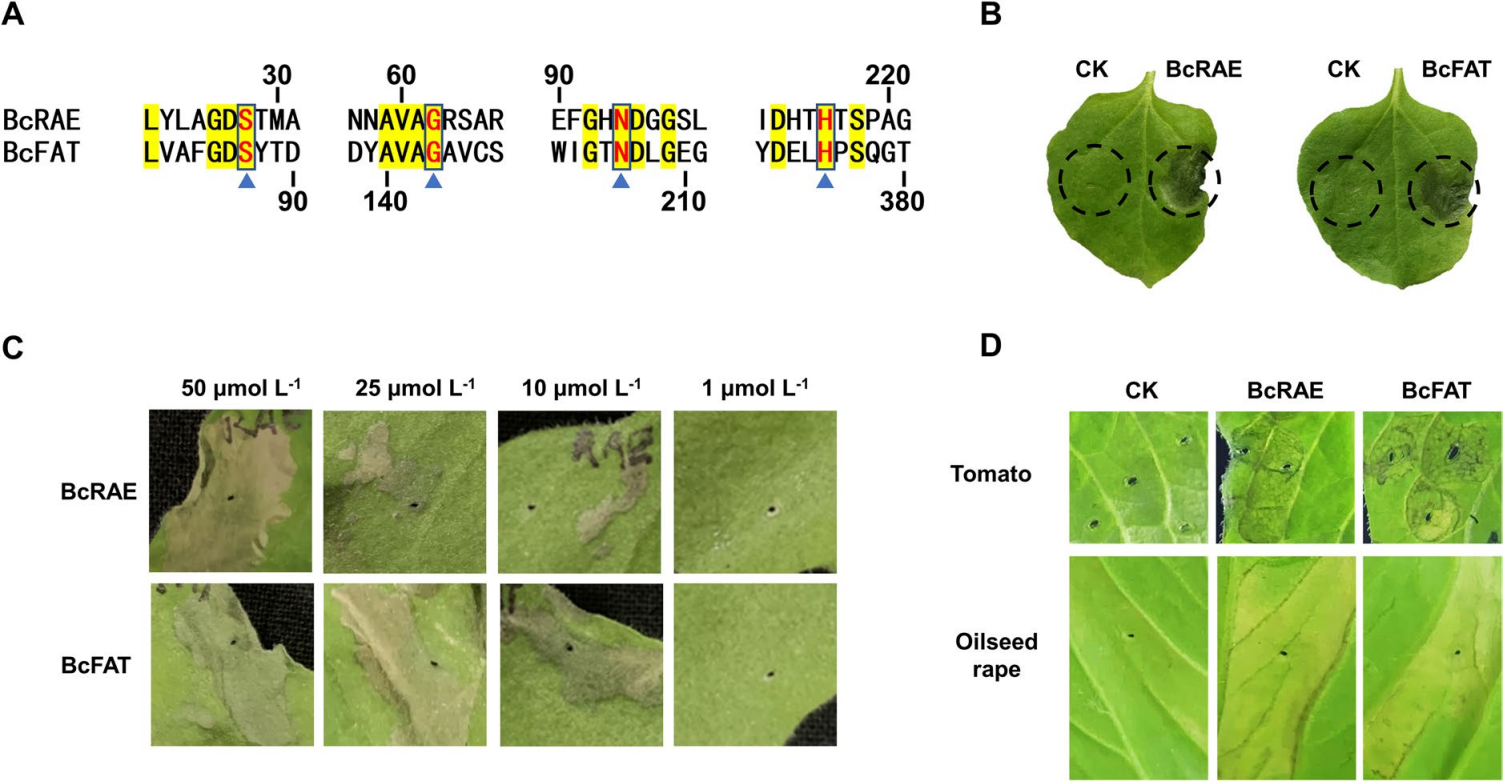
BcRAE and BcFAT induced cell death in plants. **A** Protein sequence alignment of BcRAE and BcFAT conserved motifs. Identical residues are highlighted with a yellow background. Red letters marked with blue triangles indicate the conserved residues Ser, Gly, Asn, and His in the SGNH hydrolases. The numbers indicate the position of the amino acid in the protein sequence. **B** Cell death symptoms of tobacco caused by BcRAE or BcFAT. Tobacco leaves were infiltrated with 50 μM BcRAE or BcFAT. Photos were taken 3 d after infiltration. Black dashed circles indicate infiltrated sites. **C** Effect of protein concentration on the cell death-inducing activities of BcRAE and BcFAT. For each treated leaf, the protein-infiltrated area was similar in size. Symptoms were photographed 5 d after infiltration. **D** Effect of BcRAE and BcFAT on tomato and oilseed rape leaves. Tomato and oilseed rape leaves were infiltrated with 50 μM proteins. Images were taken 5 d after infiltration

By screening candidate proteins, two novel CDIPs, rhamnogalacturonan acetylesterase (BcRAE, BCIN_02g07100) and fatty acyltransferase (BcFAT, BCIN_07g03280), were identified. The functional analysis of BcRAE and BcFAT showed that they belong to the SGNH hydrolase superfamily. The conserved residues of BcRAE and BcFAT are marked in Fig. 4A. As shown in Fig. 4B, BcRAE and BcFAT induced necrosis in tobacco leaves 3 d after infiltration, while CK protein had no apparent impact. Therefore, BcRAE and BcFAT were chosen for further mechanism research. To investigate the effect of protein concentration on the cell death-inducing activities of BcRAE and BcFAT, we adjusted the protein concentration to 1–50 μM followed by tobacco leaf infiltration. The results showed that 50 μM BcRAE caused intense necrosis. When the protein concentration was reduced to 25 μM and 10 μM, the cell death symptoms gradually weakened, and 1 μM BcRAE failed to cause cell death. BcFAT, at concentrations of 50 and 25 μM, induced intense necrosis. When the concentration was low to 10 μM, the necrosis symptoms were weakened, and 1 μM BcRAE failed to cause cell death (Fig. 4C). Moreover, we investigated the effect of BcRAE and BcFAT on tomato and oilseed rape leaves. As shown in Fig. 4D, BcRAE and BcFAT also triggered cell death in tomato and oilseed rape leaves. These results indicate that BcRAE and BcFAT can induce cell death in various plants, and the cell death-inducing activities of BcRAE and BcFAT are associated with protein concentration.

### BcRAE and BcFAT are highly expressed during infection

**Fig. 5:**
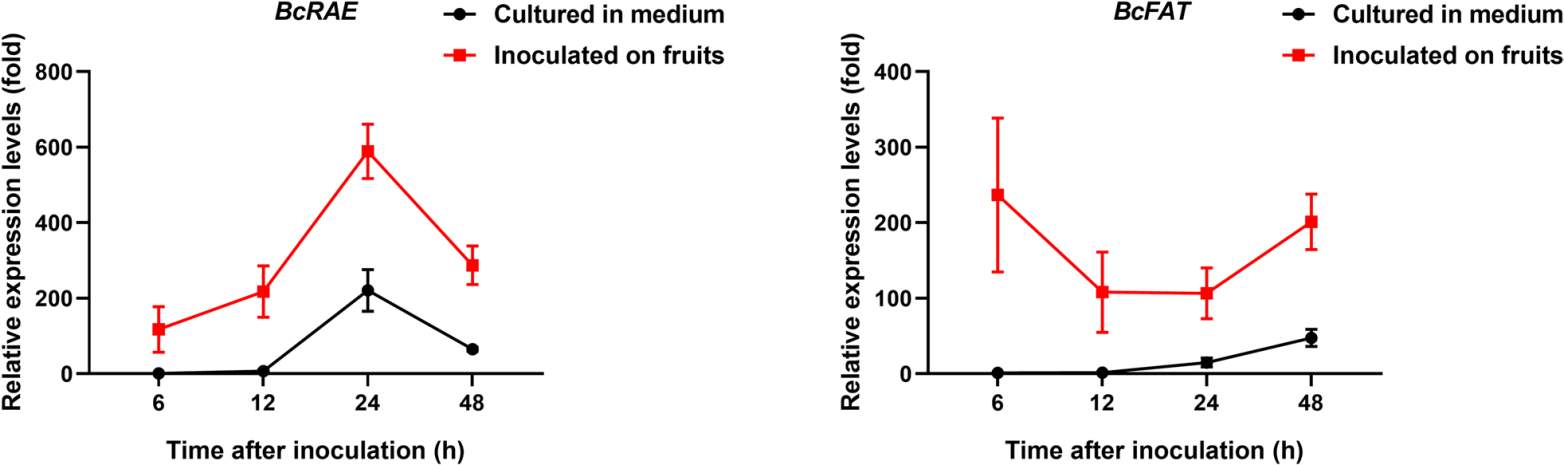
*BcRAE* and *BcFAT *are highly expressed during infection. *B. cinerea* conidia were inoculated on tomato fruits and in PDB medium. Samples were collected 6, 12, 24, and 48 h after inoculation. qRT-PCR analysis was performed to measure the expression levels of *BcRAE *and *BcFAT*. *BctubA* (BCIN_01g08040) served as the reference gene, and the gene expression level of *B. cinerea* cultured in PDB for 6 h was set to 1. The data, presented as the mean ± standard deviation (SD), represent three independent experiments

To investigate the expression patterns of *BcRAE* and *BcFAT*, we performed qRT-PCR to detect the relative expression levels of *BcRAE* and *BcFAT* at various time points during *B. cinerea* development and infection. As shown in Fig. 5, during the development of *B. cinerea* cultured in the medium, the expression level of *BcFAT* gradually increased, and the expression level of *BcRAE* peaked at 24 h post-inoculation (hpi) and then decreased. Moreover, both *BcRAE* and *BcFAT* exhibited elevated expression levels throughout the infection process (Fig. 5). Specifically, the expression of *BcRAE* during infection showed a similar trend to that during culture. On the other hand, the expression of *BcFAT* during infection increased sharply at 6 hpi, then gradually weakened during 6–24 hpi, and rose during 24–48 hpi. These results indicate that the expression of both BcFAT and BcRAE is induced during infection and suggest the potential involvement of *BcRAE* and *BcFAT* in the infection process of *B. cinerea*.

### BcRAE and BcFAT induce cell death depending on their enzymatic activities

**Fig. 6:**
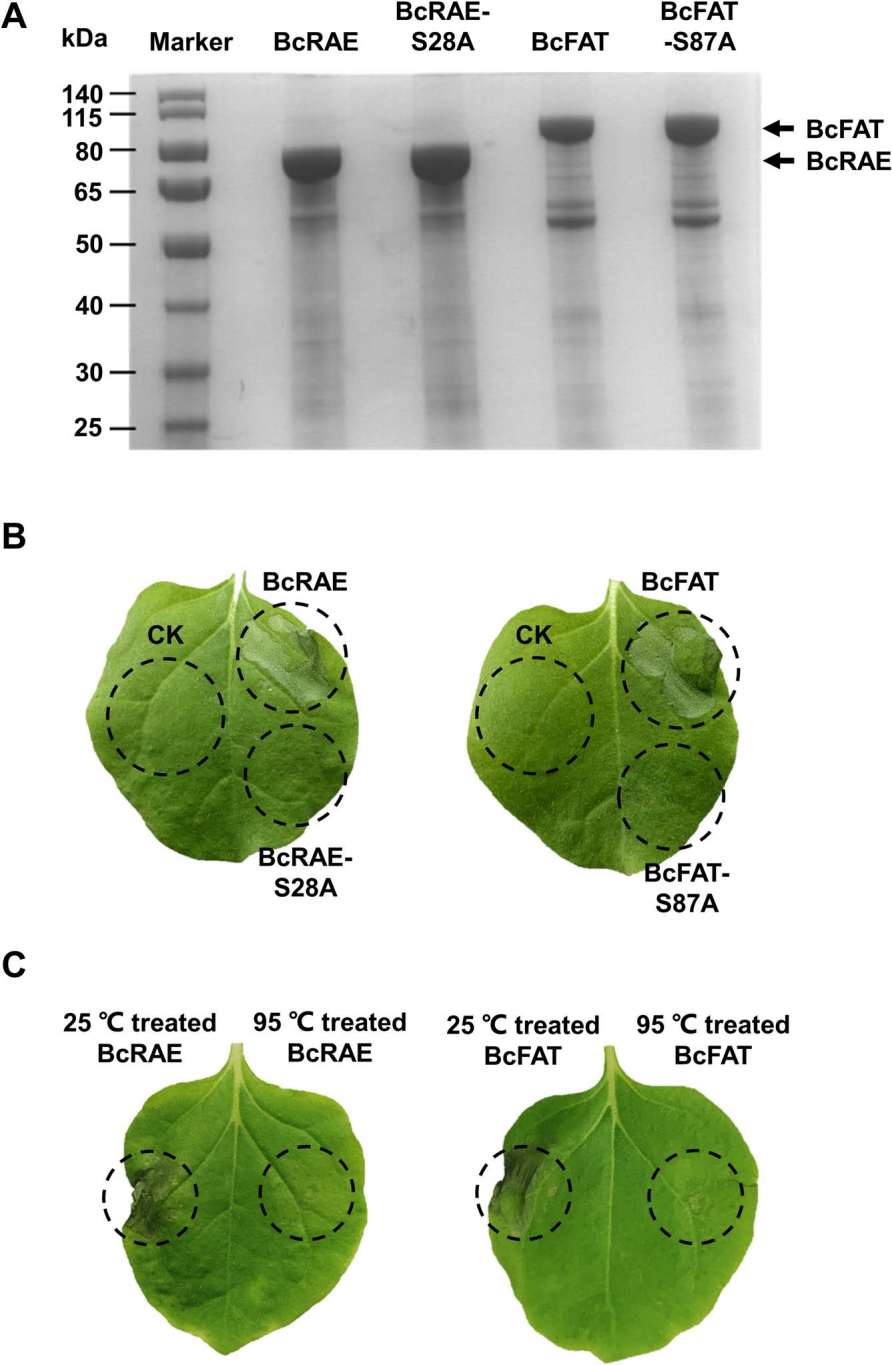
BcRAE and BcFAT induce cell death depending on SGNH hydrolase activity. **A** SDS-PAGE analysis of native and mutant proteins. The positions of the target proteins were marked with black arrows. **B** Treatment of tobacco leaves with 50 μM CK, native BcRAE and BcFAT, and mutant proteins. BcRAE-S28A: the Ser28 residue of BcRAE was mutated to Ala. BcFAT-S87A: the Ser87 residue of BcFAT was mutated to Ala. Photos were taken 3 d after treatment. Black dashed circles indicate infiltrated sites. **C** Effect of high-temperature denaturation on the cell death-inducing activities of BcFAT and BcRAE. Prokaryotically expressed proteins were incubated at 95 ℃ for 15 min and infiltrated into tobacco leaves, and proteins treated at 25 ℃ were infiltrated as the control. Photos were taken 3 d after infiltration. Black dashed circles indicate infiltrated sites

The SGNH hydrolase catalyzes the cleavage of the ester bonds, in which the conserved Ser residue is the nucleophile and proton donor. The Ser residue also participates in the formation of oxyanion holes and the catalytic triad, so it plays a crucial role in catalysis (Mlgaard et al. 2000; Akoh et al. 2004; Lescic Asler et al. 2017). To test whether the conserved Ser residues of BcRAE and BcFAT are needed for the induction of necrosis, we performed site-directed mutagenesis of Ser residues to Ala. Residues Ser28 of BcRAE and Ser87 of BcFAT were mutated to Ala. The mutant proteins were expressed in *E. coli* (Fig. 6A) and infiltrated into tobacco leaves. The CK and wild-type BcRAE and BcFAT proteins served as negative and positive controls, respectively. As shown in Fig. 6B, the cell death activities of the mutant proteins BcRAE-S28A and BcFAT-S87A were abolished. We also investigated the effect of high-temperature denaturation on the cell death-inducing activities of BcFAT and BcRAE. The result showed that high-temperature denaturation abolished the cell death-inducing activities of both BcFAT and BcRAE (Fig. 6C). These results indicate that the induction of cell death by BcRAE and BcFAT depends on their enzymatic activities.

### Knockout of BcRAE and BcFAT does not affect the growth and virulence of *B. cinerea*

To investigate the effects of BcRAE and BcFAT on the development and virulence of *B. cinerea*, we generated single and double knockout mutants of *BcRAE* and *BcFAT* (*△BcRAE-1*, *△BcRAE-2*, *△BcFAT-1*, *△BcFAT-2*, *△BcRAE/BcFAT-1* and *△BcRAE/BcFAT-2*). No significant difference was found in the colony morphology and growth rate between the mutants and the wild type (Fig. S3A). In addition, there was no significant difference in virulence between the mutant and the wild type (Fig. S3B). These results suggest that there may be functional redundancy of BcRAE and BcFAT with other SGNH family members or CDIPs in *B. cinerea*.

### BcRAE and BcFAT induce resistance of plants to *B. cinerea* independent of their enzymatic activities

**Fig. 7:**
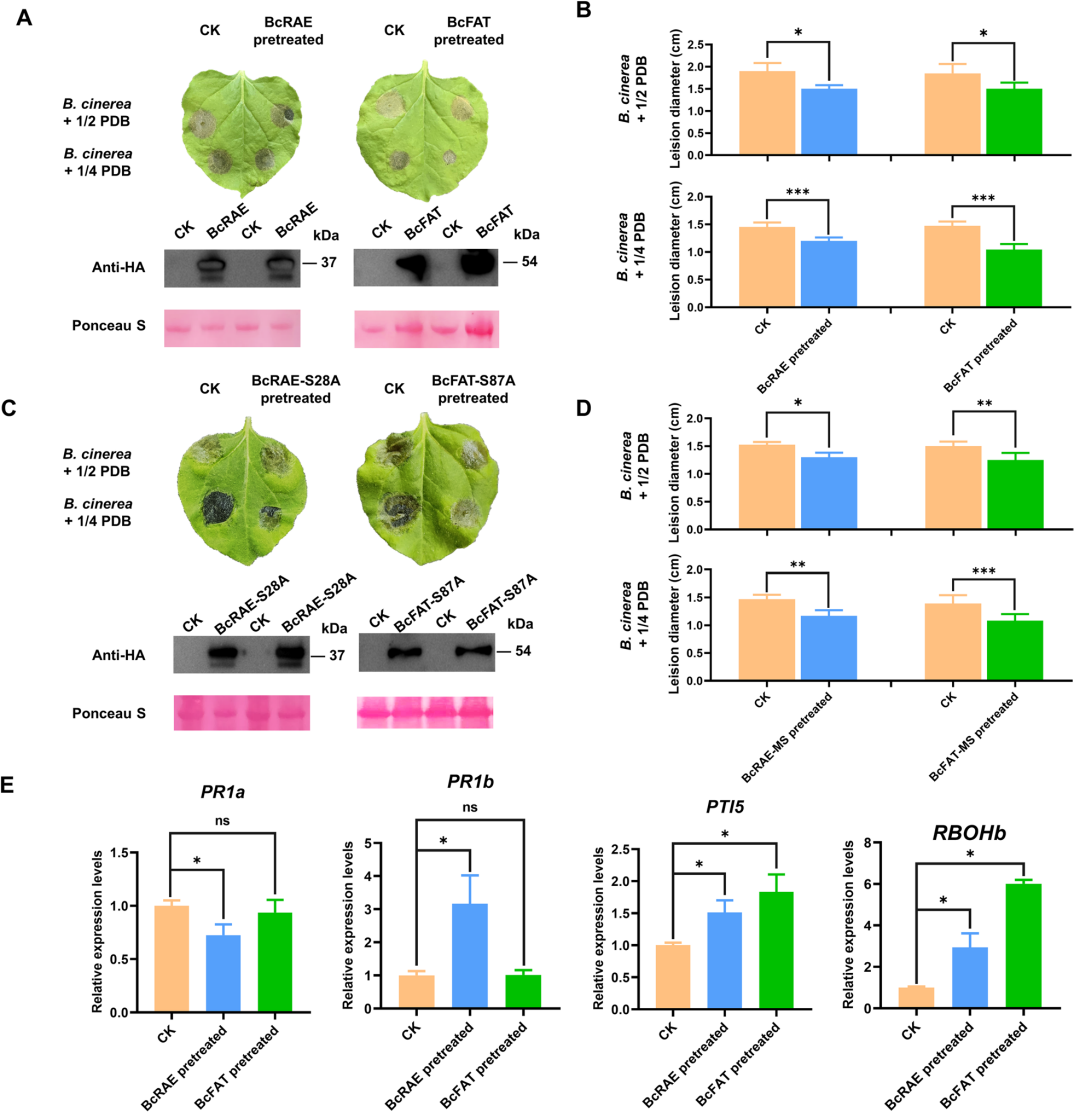
BcRAE and BcFAT induce resistance in *N. benthamiana*. **A** and **C** Phenotypes of plant resistance to *B. cinerea* induced by transiently expressed proteins. *BcFAT*, *BcRAE* **A** and their site-mutated proteins **C** were transiently expressed in N. benthamiana, and the Agrobacterium harboring the empty PVX vector was used as the control. The treated tobacco was kept in the culture room for three days, followed by inoculation of B. cinerea spores resuspended in PDB of different dilutions on the Agrobacterium-infiltrated site. Protein expression was detected by WB, and Ponceau S staining served as the loading control. The inoculated plants were kept in a humid chamber at 22 °C. Photographs were taken 60 h after inoculation. Each assay contained at least nine leaves, and the experiment was repeated three times. **B** and **D** Statistics of colony diameters. Colony diameters of *B. cinerea* grown on BcFAT, BcRAE **B** and their site-mutated proteins **D** pretreated tobacco were measured 60 h after inoculation. Data from three independent experiments are presented as the mean ± SD. Asterisks represent a significant difference (Tukey test, *, *P* < 0.05, **, *P* < 0.01, ***, *P* < 0.001); E N. benthamiana leaves were infiltrated with 50 μM of control protein, *BcFAT*, and *BcRAE*. After 24 h of growth in the culture room, the treated leaves were picked for RNA extraction. qRT-PCR was used to analyze the expression levels of *NbPR1a*, *NbPR1b*, *NbPTI5*, and *NbRBOHb*. Nbef1α was used as the reference gene. Data from three independent experiments are presented as the mean ± SD. Asterisks represent a significant difference. (Tukey test, *P* < 0.05)

Previous studies have shown that some SGNH family esterases/lipases in plants have different regulatory effects on plant immunity (Oh et al. 2005; Hong et al. 2008; Kwon et al. 2009; Lee et al. 2009; Kim et al. 2013; Gao et al. 2017). To determine whether BcRAE and BcFAT induce plant immune responses, we transiently expressed BcRAE and BcFAT by infiltrating Agrobacterium into tobacco leaves (no obvious symptoms, data not shown). WB results verified the expression of proteins in tobacco (Fig. 7A). We further inoculated *B. cinerea* spores on infiltrated sites three days after infiltration. The results showed that the lesion sizes of the leaves pretreated with BcRAE and BcFAT were significantly smaller (*P* < 0.05) than those of the control (Fig. 7A and B). Moreover, BcFAT-pretreated tobacco leaves had smaller lesion sizes after inoculation with *B. cinerea* spores resuspended in PDB diluted 1:4 with water compared to control and BcRAE. In addition, we transiently expressed site-mutated BcFAT and BcRAE in tobacco. Interestingly, the transient expression of site-mutated proteins still significantly enhanced plant resistance to *B. cinerea* (Fig. 7C and D). These results indicate that BcRAE and BcFAT induce plant resistance to *B. cinerea* independent of their enzymatic activities, which differs from their cell death-inducing activities.

To further analyze the effect of BcFAT and BcRAE on defense-related genes, we infiltrated tobacco leaves with 50 μM BcFAT, BcRAE, and CK protein. The expression levels of *NbPR1a, NbPR1b*, *NbPTI5*, and *NbRBOHb* at 24 hpi were detected by RT-qPCR. The results showed that BcRAE significantly induced the expression of *NbPR1b*, *NbPTI5*, and *NbRBOHb* but inhibited the expression of *NbPR1a.* BcFAT induced the expression of *NbPTI5* and *NbRBOHb* but had no significant effect on *NbPR1a* and *NbPR1b* (Fig. 7E). These results suggest that BcRAE and BcFAT induce plant immunity-related gene expression and enhance tobacco resistance to *B. cinerea*.

## Discussion

Unlike biotrophic pathogens that absorb nutrients from living cells, necrotrophic pathogens generally kill host cells to obtain nutrients. As a necrotrophic fungus, *B.cinerea* has long been thought to kill hosts crudely by secreting numerous cell wall-degrading enzymes, toxins, and other metabolites. However, recent studies have shown that the interaction between *B. cinerea* and host plants is complex and delicate (Bi et al. 2023). Once *B. cinerea* is in contact with host cells, they first face the trouble of obtaining nutrients from living cells and withstanding plant defense. Therefore, the early stage of infection is critical for successful colonization. According to the infection model proposed by Shlezinger et al. (2011), the anti-apoptotic machinery protects *B. cinerea* from host-induced cell death, and *B. cinerea* secretes various necrosis-inducing factors to establish small infection zones. The surviving fungal cells develop and accumulate sufficient fungal biomass for further lesion expansion. Nevertheless, studies of *Sclerotinia sclerotiorum*, the sister species of *B. cinerea*, suggested a short biotrophic phase in its early infection stage (Kabbage et al. 2013). Veloso and van Kan (2018) proposed an alternative model. According to this model, the balance between apoptosis and autophagy in *B. cinerea* and the plant host determines the result of colonization. In the biotrophic phase, *B. cinerea* produces autophagy-suppressing molecules to suppress host autophagic cell death. When fungi accumulate sufficient biomass in host tissues, plant apoptosis triggered by fungal CDIPs and toxins replaces the suppression of autophagy, resulting in the death of plant tissues and the spread of disease.

Although whether the biotrophic phase is included in the early stages of *B. cinerea* infection remains unclear, a consensus exists between the two models that CDIPs play an essential role in the early stage of colonization. Therefore, the study of CDIPs is of great significance for analyzing the interaction between *B. cinerea* and plants. Previously reported secretomes of *B.cinerea* showed that more than 500 proteins are secreted during infection, including CWDEs, proteases, and many proteins with unknown functions (Shah et al. 2009a; Espino et al. 2010; Zhu et al. 2017). However, only a few CDIPs have been reported (Zhu et al. 2017, 2022; Yang et al. 2018b; Bi et al. 2021; Jeblick et al. 2023). Recently, Leisen et al. (2022) generated multiple knockout mutants lacking 12 reported CDIPs through the CRISPR/Cas9 system and found that the secretome of the mutant strains still retained substantial phytotoxic activity, indicating that there are still unknown CDIPs triggering cell death. Therefore, it is necessary to further screen CDIPs from the remaining large number of secreted proteins, and an efficient screening system is needed for high-throughput CDIP screening.

There are three commonly used protein expression systems: prokaryotic, eukaryotic, and *Agrobacterium*-mediated transient expression systems. The *Agrobacterium*-mediated transient expression system is convenient and efficient. Therefore, it has been widely used previously to characterize secreted proteins (Takken et al. 2000). The eukaryotic expression system has the ability to perform post-translational modifications, making the combined protein closer to the original structure of the native protein. Therefore, it is also used in some studies on protein functional characterization. The prokaryotic expression system has high expression efficiency. Although some studies have verified its feasibility in characterizing CDIP, the frequent formation of inclusion bodies and non-uniform expression conditions limit its application in large-scale CDIP screening (Zhang et al. 2017; Zhu et al. 2017). It has been suggested that excessive and rapid expression and incorrect disulfide bond pairing are the main reasons for forming inclusion bodies (Paraskevopoulou and Falcone 2018). Several strategies have been proposed in recent years to solve the formation of inclusion bodies, such as optimizing strains, lowering the temperature, and co-expressing molecular chaperones (Rosano and Ceccarelli 2014). Since most of the secreted proteins have small molecular weights, they can be synthesized rapidly in *E. coli* and are prone to form inclusion bodies. Here, we explored different expression conditions to optimize the prokaryotic expression system for screening CDIP. Co-expression of CDIP with the molecular chaperone TF at 15 ℃ efficiently solved the CDIP inclusion body formation problem (Fig. S1). TF is derived from *E. coli* with outstanding solubility and has been proposed as a folding catalyst, effectively preventing the aggregation of recombinant proteins (Nishihara et al. 2000). Furthermore, we verified that high concentrations of TF have no obvious impact on plants, which suggests that the optimized prokaryotic expression system is suitable for CDIP screening (Fig. 1).

To determine the expression system for large-scale CDIP screening, we further compared the effectiveness of different expression systems for validating reported CDIPs in *B. cinerea*. The results showed that the previously reported BcSPL1 expressed by the transient expression system failed to induce cell death, possibly because the transiently expressed protein level was insufficient. Therefore, the *Agrobacterium*-mediated transient expression system may miss some CDIPs during large-scale screening. Although the eukaryotic expression system showed excellent screening effectiveness, it is unsuitable for large-scale screening due to its time-consuming and labor-intensive properties (Fig. 2). Consequently, we chose the prokaryotic expression system as the final screening system.

Through the prokaryotic expression system, we validated the cell death-inducing activity of 55 proteins, mainly cell wall-degrading enzymes, immune-associated proteins, proteases, and oxidoreductases. Among the 55 candidate proteins, two SGNH family CDIPs, BcRAE and BcFAT, were identified. The SGNH hydrolase superfamily (once called the GDSL family), named after the four conserved residues serine, glycine, asparagine, and histidine, is widely found in prokaryotes and eukaryotes with a broad range of substrates (Lescic Asler et al. 2017). The SGNH hydrolase superfamily comprises a diverse range of carbohydrate-modifying enzymes, including but not limited to carbohydrate esterase (Anderson et al. 2022). The crystal structures of SGNH family proteins have been extensively studied (Mlgaard et al. 2000; Lescic Asler et al. 2017; Anderson et al. 2022). SGNH hydrolases adopt an alpha/beta/alpha-sandwich fold. Enzymes containing this domain act as esterases and lipases but have little sequence homology to true lipases. Furthermore, our results showed that *BcRAE* and *BcFAT* were highly expressed during infection, suggesting a potential role of BcRAE and BcFAT in *B. cinerea*-plant interactions (Fig. 5). Notably, signal peptide prediction indicates that BcFAT lacks a signal peptide. Nevertheless, BcFAT has been identified in multiple secretome studies (Shah et al. 2009a, 2009b; Espino et al. 2010; Zhu et al. 2017; Liu et al. 2023), suggesting that BcFAT may be secreted to the extracellular space through an unconventional secretion pathway. Besides the classical protein secretion pathway mediated by signal peptides, several unconventional protein secretion routes have been reported in eukaryotes (Zhang and Schekman 2013; Rabouille 2017). BcGS1, a previously reported CDIP without a signal peptide, was identified in the secreted proteins of *B. cinerea* (Zhang et al. 2015). Additionally, several virulence factors in other fungi have been reported to be secreted to the extracellular space through non-classical secretion pathways (Artier et al. 2018; Li et al. 2019). Previous studies have reported that SGNH hydrolases in plants play different roles in plant immunity. *Arabidopsis* GDSL-like lipase 1 (GLIP1) has been reported to play an essential role in plant immunity by regulating local and systemic resistance in plants through the ethylene pathway. Overexpression of *GLIP1* in *Arabidopsis* enhanced plant resistance, such as *Alternaria brassicicola*, *Erwinia carotovora*, and *Pseudomonas syringae* (Oh et al. 2005; Kwon et al. 2009; Kim et al. 2013). However, silencing *CaGLIP1* in pepper increased resistance to *Xanthomonas campestris*, and overexpression of *CaGLIP1* in *Arabidopsis* reduced plant resistance to *P. syringae* and *Hyaloperonospora parasitica* (Hong et al. 2008). Moreover, OsGLIP1 and OsGLIP2 were reported to negatively regulate rice resistance to *Xanthomonas oryzae* and *Magnaporthe oryzae* by modulating lipid metabolism (Gao et al. 2017).

It has been reported that the necrosis-inducing activity of some CDIPs is dose-dependent (Frías et al. 2016; Zhu et al. 2022). Our results show that BcFAT and BcRAE at 50 μM triggered intense cell death. Symptoms were weakened with decreasing protein concentrations (Fig. 4). When the protein concentration was as low as 1 μM, both failed to trigger necrosis, suggesting that the necrosis-inducing activity of BcFAT and BcRAE is dose-dependent, which also explains the inability of transiently expressed BcFAT and BcRAE to trigger necrotic symptoms. Some CDIPs have been reported to induce cell death independent of their enzymatic activities. For example, Bcxyn11 triggers necrosis in tobacco, and its necrosis-inducing activity does not depend on its xylanase activity (Noda et al. 2010). Similar results for BcXYG1 and BcCRH1 were reported (Zhu et al. 2017; Bi et al. 2021). We also examined whether the cell death-inducing activities of BcFAT and BcRAE depended on their SGNH hydrolase activities. The results showed that the cell death-inducing activities of both BcFAT and BcRAE were abolished when the conserved catalytic residue Ser was mutated to Ala. In addition, high-temperature denaturation also abolished the cell death-inducing activities of both BcFAT and BcRAE. These results suggest that enzyme activities are indispensable for BcRAE and BcFAT-induced cell death. Since both BcRAE and BcFAT target cell wall components, the degradation products of the enzymes may function as damage-associated molecular patterns (DAMPs) to trigger cell death in plants. Similar results have been reported before. For example, It has been reported that Mocel12A/B of *M. oryzae* does not trigger the immune response by itself, but oligosaccharides derived from the degradation of the cell wall by Mocel12A/B activate the immune response in plants (Yang et al. 2021). The effect of BcFAT and BcRAE degradation products on plant immunity needs further study. Moreover, our results show that the single and double knockout of BcFAT and BcRAE did not affect the growth and virulence of *B. cinerea* (Fig. S3). This result is unsurprising because there are four homologous proteins of BcFAT and two homologous proteins of BcRAE in *B. cinerea*. We speculated that the functional redundancy among homologous proteins or CDIPs might lead to the absence of a significant phenotype in these mutant strains. Similar results have been reported for BcXYG1, BcCRH1, and BcIEB1 (Frías et al. 2016; Zhu et al. 2017; Bi et al. 2021).

During long-term co-evolution with pathogens, plants have developed a sophisticated immune system to resist the attack of pathogens. Conserved pathogen-associated molecular patterns (PAMPs) from pathogens and DAMPs from plants are recognized by plant pattern recognition receptors (PRRs) embedded in the plasma membrane and activate pattern-triggered immunity (PTI). Specific adapted pathogens further secrete effectors to suppress PTI, while intracellular disease resistance proteins specifically recognize effectors and activate effector-triggered immunity (ETI) (Xu et al. 2022). Here, we found that *BcFAT* and *BcRAE* are highly expressed during early infection. Abundantly expressed BcFAT and BcRAE are sufficient to induce cell death, which depends on their enzymatic activities. Nevertheless, transient expression of BcRAE and BcFAT could not induce cell death but enhanced plant resistance to *B. cinerea*. Moreover, transient expression of site-mutated BcRAE and BcFAT could still induce plant resistance against *B. cinerea*. These results indicate that the resistance-inducing activities of BcFAT and BcRAE are independent of their enzymatic activities, which differs from their cell death-inducing activities. We speculate that BcFAT and BcRAE play a two-faced role during the plant-*B. cinerea* interaction. During early infection, *B. cinerea* secretes abundant BcFAT and BcRAE to kill plant cells by destroying cell walls. As a response, plants evolve corresponding receptors to recognize them and activate plant immunity to limit the infection of *B. cinerea*. Therefore, exploring BcRAE and BcFAT recognition proteins in plants would be interesting. Analysis of immune-related gene expression showed that BcRAE induces the expression of *NbPR1b* but suppresses *NbPR1a*. *PR1* is commonly considered a marker of systemic acquired resistance (Vleeshouwers et al. 2000; Boyle et al. 2009; Frackowiak et al. 2019). Unlike BcRAE, BcFAT had no significant effect on *NbPR1a/b* expression, suggesting that BcFAT may act through a different pathway. Both BcFAT and BcRAE resulted in the upregulation of *NbPTI5* and *NbRBOHb*. *PTI5* is a marker of the PTI response and regulates a series of defense-related genes downstream (Nguyen et al. 2010). The activation of *NbRBOHb* is associated with a reactive oxygen species (ROS) burst, an early event of the plant immune response (Irieda et al. 2018). The qRT-PCR results are consistent with enhanced resistance in plants. These results suggest that BcRAE and BcFAT may trigger the plant PTI response and induce the expression of downstream defense genes and the burst of ROS, thus enhancing resistance to *B. cinerea*.

In conclusion, we compared different protein expression systems and suggested that the optimized prokaryotic expression system is suitable for high-throughput CDIP screening. With the help of the prokaryotic expression system, two novel SGNH family CDIPs, BcRAE and BcFAT, were identified. We also preliminarily resolved the mechanism of BcFAT and BcRAE. Our results provide new targets for further study of *B. cinerea*-plant interactions and lay a theoretical foundation for controlling *B. cinerea*.

## Methods

### Fungal strains and plant materials

*Botrytis cinerea* (B05.10) was used as the wild-type and recipient stain to generate mutant strains. All strains were cultured in continuous light on potato dextrose agar (PDA) medium at 22 °C. *Nicotiana benthamiana* was cultivated in a culture room at 23 °C with 16 h of daily illumination.

### Candidate-secreted protein selection and plasmid construction

Candidate-secreted proteins were selected from previously reported secretomes of *B. cinerea* (Shah et al. 2009a; Shah et al. 2009b; Espino et al. 2010; Li et al. 2012; Zhu et al. 2017; Li et al. 2020; Liu et al. 2023). The candidate gene sequences were obtained from NCBI (http://www.ncbi.nlm.nih.gov) and EnsemblFungi (http://fungi.ensembl.org/Botrytis_cinerea/Info/Index). The protein function and signal peptide information were retrieved from EnsemblFungi.

Total RNA was extracted from *B. cinerea* mycelia using TRNzol reagent (Tiangen, China) as previously described (Zhang et al. 2021b). cDNA was synthesized using the PrimeScript RT reagent Kit (Takara, Japan) following the manufacturer’s instructions. The ORF sequences of target genes without signal peptides were amplified from the synthesized cDNA. The linearized vectors pPIC9K and pCold-TF were used to construct eukaryotic and prokaryotic expression vectors, respectively. For the construction of *Agrobacterium*-mediated transient expression vectors, the *Arabidopsis PR3* signal peptide was fused to the N-terminus of the PVX-3 × HA vector according to the description of Zhu et al. (2017) to form PVX-SP-3 × HA vector that enables proteins to be secreted to the apoplast. The PVX-SP-3 × HA vector was linearized with the restriction enzymes ClaI and NotI. The amplified fragments were fused with the linearized expression vectors by homologous recombination using an SE Seamless Cloning and Assembly Kit (Zoman, China). All constructed vectors were confirmed by sequencing. The primers used in this paper are listed in Supplementary Table S1.

### Protein expression and cell death-induction assay

Protein expression in the eukaryotic system was conducted following the protocol by Zhang et al. (2017) with minor adjustments. In brief, the pPIC9K vector fused with the candidate gene was linearized by SalI and then transformed into *Pichia pastoris* GS115, and an empty pPIC9K vector was introduced as the control. A Mut^+^ (methanol utilization plus phenotype) transformant carrying muti-copy expression cassettes was screened by methanol and geneticin (G418) for subsequent protein expression. The transformant was cultured in BMMY medium with 0.5% methanol for 48 h. The crude protein was precipitated from the supernatant of the liquid culture by saturated ammonium sulfate solution. The 6 × His labeled recombinant protein was then purified using Ni–NTA agarose (Qiagen, USA) and verified by gel electrophoresis. Finally, with the help of Amicon Ultra Centrifugal Filter Units (Merck Millipore, Germany), verified proteins were concentrated, and the buffer was exchanged with PBS buffer (0.01 M phosphate buffer solution, pH 7.4).

The prokaryotic expression of proteins was conducted following Xing et al. (2021), with slight modifications. Constructed pET30a or pCold-TF plasmids were introduced into *Escherichia coli* strain *Rosetta*. Positive colonies were incubated in 10 mL ampicillin-supplemented LB broth medium at 37 °C overnight. The overnight-incubated *E. coli* was added to 1 L of fresh LB broth containing ampicillin and cultured at 37 °C until the OD_600_ reached 0.5. IPTG induced the culture with a final concentration of 0.5 mM. For expression with the pET30a vector, the culture was induced at 37 °C for 5 h or 15 °C for 16 h. For expression with the pCold-TF vector, the culture was incubated at 15 °C for 16 h. *E. coli* cells were collected, resuspended, and sonicated on ice until the solution was clear. The lysate was centrifuged at 8000 g at 4 °C for 20 min. The supernatant and precipitate were collected separately for SDS-PAGE. Protein purification, verification, concentration, and buffer replacement were the same as described in the eukaryotic expression system. Protein concentrations were measured by Bradford assay with a BSA standard curve (Bradford 1976).

*Agrobacterium*-mediated transient expression was performed according to Wang et al. (2011). The recombinant plasmids were introduced into Agrobacterium *tumefaciens* strain GV3101 (pJIC SA_Rep) by electroporation, and the empty PVX-SP-3 × HA vector was transformed as the control. The positive transformants were cultured in LB broth with kanamycin (50 mg L^−1^) and rifampicin (50 mg L^−1^). Cultured Agrobacterium cells were collected, washed, and resuspended in 10 mM MgCl_2_. The OD_600_ was finally adjusted to 0.5. The suspension was then incubated at room temperature for 1 h before infiltration.

For the cell death induction assay, the Agrobacterium cell suspension, 50 μM purified prokaryotically expressed protein, and 50 μM purified eukaryotically expressed protein were infiltrated into plant leaves. For high-temperature denaturation, proteins were incubated at 95 ℃ for 15 min. Each assay contained at least nine leaves. Cell death symptoms were monitored and recorded 3 to 7 d after infiltration.

### Gene expression pattern analysis

For the gene expression pattern analysis, *B. cinerea* conidia (2 × 10^5^ spores mL^−1^) were inoculated on tomato fruits or into PDB medium. Samples were collected at 6, 12, 24, and 48 hpi, and RNA extraction was subsequently performed. The qRT-PCR was utilized to detect the relative expression levels of genes (Liu et al. 2023). *BctubA* (BCIN_01g08040) served as the reference gene, and the gene expression level of *B. cinerea* cultured in PDB for 6 h was set to 1.

### Site-directed mutagenesis

The process of site-directed mutagenesis was carried out using the M5 HiPer Site-Directed Mutagenesis Kit (Mei5bio, China) following the manufacturer’s instructions. The pCold-TF plasmids harboring the ORF of *BcFAT* or *BcRAE* were used as templates to obtain site-directed mutants. The primer pairs for mutagenesis were designed using an online design tool (http://ps.biocloud.org.cn/). Site-mutated gene fragments were amplified from mutated pCold vectors and then homologously recombined into the PVX vector to construct transient expression vectors of site-mutated proteins.

### Generation of knockout mutants and phenotype assay

The knockout mutant generation and phenotype assay followed a previously described procedure (Li et al. 2020). The gene knockout of *B. cinerea* was based on the gene replacement strategy. In brief, the hygromycin resistance cassette flanked by the upstream and downstream regions of *BcFAT* or *BcRAE* was transformed into protoplasts of wild-type *B. cinerea*. Hygromycin B was used to select positive transformants, followed by single-spore isolation to isolate homokaryons. The homokaryons were verified by flank-spanning PCR. The *∆BcFAT/BcRAE* double knockout mutant was generated based on the *∆BcRAE* single knockout mutant, according to Zhang et al. (2021b). The nourseothricin resistance cassette was amplified from the pNAN-OGG vector and fused with the upstream and downstream regions of *BcFAT.* The fused fragment was transformed into protoplasts of the *∆BcRAE* mutant strain, and positive transformants were screened on PDA plates containing nourseothricin.

For growth phenotype assay, the homokaryotic mutants and wild-type were cultured on PDA medium plates at 22 °C under light. The colony diameters of cultures were measured 2 to 3 days postinoculation. The assay was repeated three times. For the pathogenic phenotype assay, spores of wild-type and mutants were inoculated on sterilized apple fruits (*Malus pumila* Mill cv. Fuji). Lesion development was monitored and recorded three days postinoculation. At least nine apples were used in each assay. The experiment contained three repeats.

### Induction of plant resistance and defense-related gene expression

To investigate the effect of BcFAT and BcRAE on plant resistance, BcFAT and BcRAE were expressed in *N. benthamiana* through the *Agrobacterium*-mediated transient expression system described above. Protein-expressing Agrobacterium and the control Agrobacterium infiltrated different sides of the same leaf. The treated tobacco was kept in the culture room for three days, followed by inoculation of 10 μL of *B. cinerea* spore suspension (2 × 10^5^ spores mL^−1^ diluted PDB) on the *Agrobacterium*-infiltrated site. WB was performed to detect the expression of proteins. The inoculated plants were kept in a humid chamber at 22 °C under the light. The lesion development was recorded 60 h postinoculation. Each assay contained at least nine leaves, and the experiment was repeated three times. *N. benthamiana* leaves were infiltrated with 50 μM control protein, BcFAT, and BcRAE to detect the defense-related gene expression. After 24 h of growth in the culture room, the treated leaves were picked for RNA extraction and qRT-PCR analysis. The experiment was repeated three times.

### Statistical analysis

Statistical differences in phenotype assay, resistance induction, and defense-related gene expression experiments were analyzed using IBM SPSS Statistics 21 software (IBM, USA). Comparisons were performed through one-way ANOVA with Tukey’s test, and a *P*-value < 0.05 was considered significant.

### Supplementary Information

**Additional file 1:** **Table S1.** Information on candidate proteins and primers used in this paper.

**Additional file 2:** **Figure S1.** Optimization of prokaryotic expression conditions.

**Additional file 3:** **Figure S2.** Expression of candidate proteins by the prokaryotic expression system.

**Additional file 4:** **Figure S3.** Phenotypes of BcFAT and BcRAE knockout mutant strains.

## Data Availability

The data will be available from the corresponding author upon reasonable request.

## Abbreviations

CDIP: Cell death-inducing protein
DAMP: Damage-associated molecular pattern
ETI: Effector-triggered immunity
GLIP: GDSL-like lipase
hpi: Hours post-inoculation
PAMP: Pathogen-associated molecular pattern
PTI: Pattern-triggered immunity
ROS: Reactive oxygen species
SD: Standard deviation
TF: Trigger factor
WB: Western blot
