# Supplementary figures and images for: Characterization of two SGNH family cell death-inducing proteins from the horticulturally important fungal pathogen Botrytis cinerea based on the optimized prokaryotic expression system
Source: Mol Hortic. 2024 Mar 7;4:9. doi: 10.1186/s43897-024-00086-3 (PMC10919021; doi:10.1186/s43897-024-00086-3)

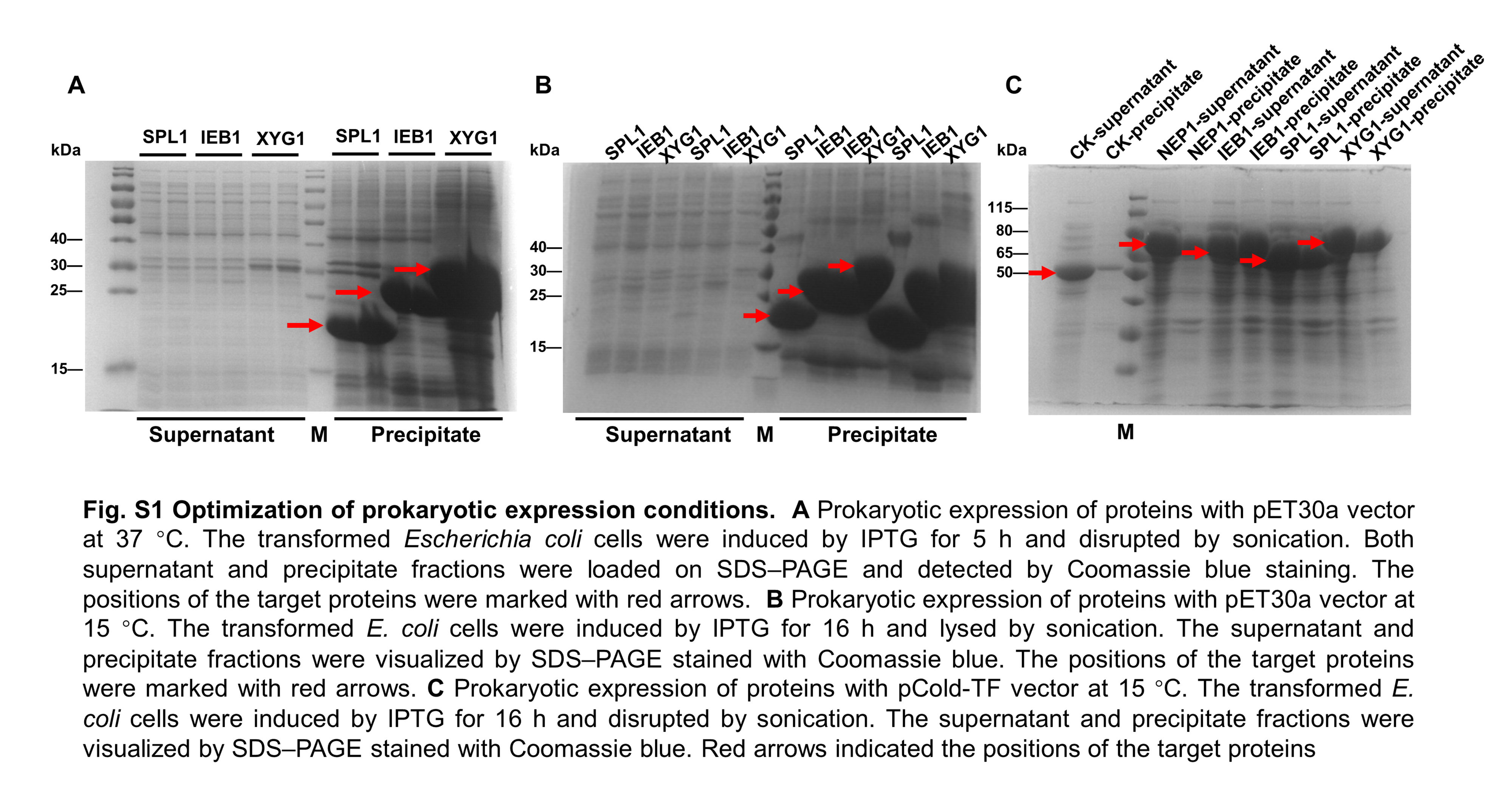

Supplement: Supplementary file 2 — Additional file 2: Figure S1. Optimization of prokaryotic expression conditions. [file 43897_2024_86_MOESM2_ESM.png]

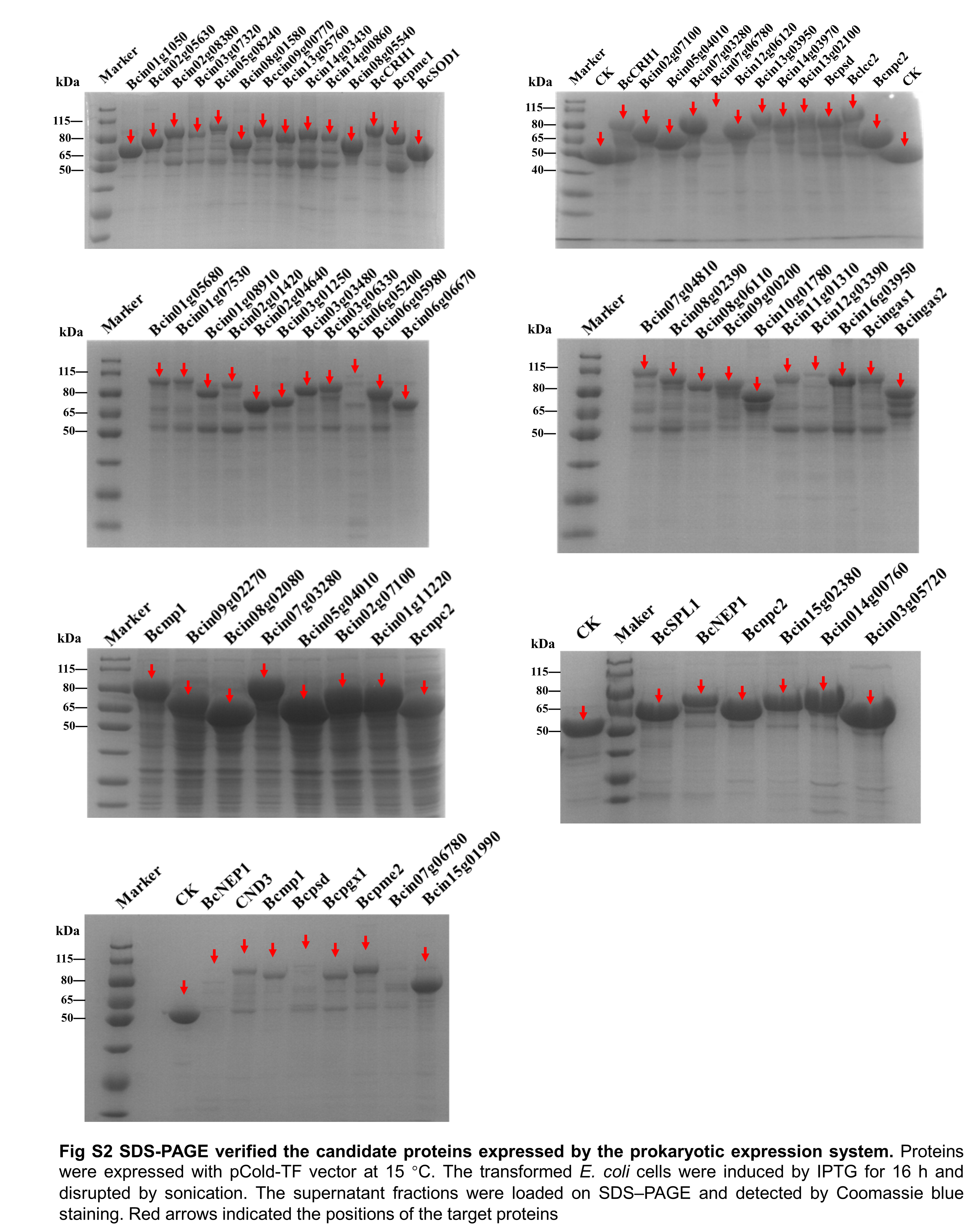

Supplement: Supplementary file 3 — Additional file 3: Figure S2. Expression of candidate proteins by the prokaryotic expression system. [file 43897_2024_86_MOESM3_ESM.png]

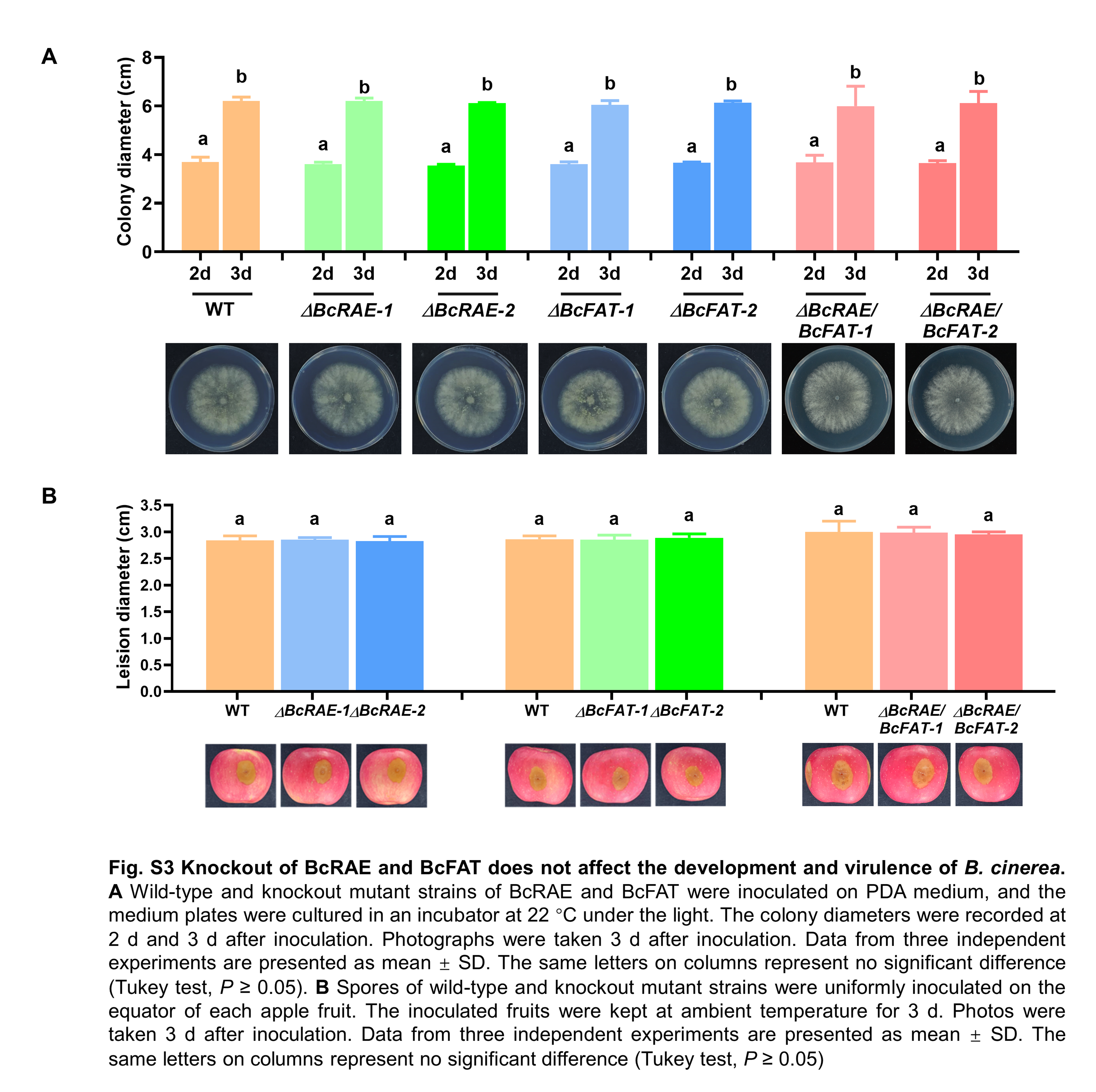

Supplement: Supplementary file 4 — Additional file 4: Figure S3. Phenotypes of BcFAT and BcRAE knockout mutant strains. [file 43897_2024_86_MOESM4_ESM.png]
